# Risk factors for biliary tract events during elective cholecystectomy waiting time after endoscopic retrograde cholangiopancreatography for choledocholithiasis

**DOI:** 10.1002/deo2.409

**Published:** 2024-08-13

**Authors:** Tatsunori Satoh, Junichi Kaneko, Shinya Kawaguchi, Yuya Ishiguro, Shinya Endo, Naofumi Shirane, Hideyuki Kanemoto, Takanori Yamada, Kazuya Ohno

**Affiliations:** ^1^ Department of Gastroenterology Shizuoka General Hospital Shizuoka Japan; ^2^ Deparment of Gastroenterology Iwata City Hospital Shizuoka Japan; ^3^ Department of Gastroenterology Japanese Red Cross Shizuoka Hospital Shizuoka Japan; ^4^ Department of Surgery Shizuoka General Hospital Shizuoka Japan

**Keywords:** biliary stent, cholangitis, cholecystectomy, cholecystitis, choledocholithiasis

## Abstract

**Objectives:**

Endoscopic lithotripsy and elective cholecystectomy, followed by endoscopic retrograde cholangiopancreatography, are the first‐line treatments for patients with common bile duct (CBD) stones (CBDS) and gallstones. However, this approach entails acute cholecystitis and recurrent cholangitis risk while patients await surgery. We aimed to identify acute cholecystitis and cholangitis risk factors during the waiting time for elective cholecystectomy.

**Methods:**

This study comprised 151 patients with CBDS combined with gallstones who underwent cholecystectomy within 90 days of the first endoscopic retrograde cholangiopancreatography at two tertiary care centers between January 2019 and October 2021.

**Results:**

The incidence of biliary tract events (acute cholecystitis, acute cholangitis, or any complications requiring unplanned cholangiopancreatography) was 28% (43 cases). In univariate and multivariate analyses, plastic stent placement as a bridge to surgery for the first treatment of CBDS was an independent risk factor for biliary tract events during the waiting time for surgery (odds ratio 4.25, *p* = 0.002). A subgroup analysis among those with plastic stent placement revealed a CBD diameter of ≤ 10 mm as an independent risk factor for acute cholecystitis (odds ratio 4.32; *p* = 0.027); a CBD diameter ≥ 11 mm was an independent risk factor for acute cholangitis and unplanned re‐endoscopic retrograde cholangiopancreatography (odds ratio 5.66; *p* = 0.01).

**Conclusions:**

Plastic stent placement for CBDS before elective cholecystectomy increases the risk of acute cholecystitis or acute cholangitis during the waiting time for elective cholecystectomy.

## INTRODUCTION

Cholecystocholedocholithiasis (CCL), the coexistence of common bile duct (CBD) stones (CBDS) with gallstones, is a common clinical disease, accounting for 8%–20% of patients with gallstones.^1^, ^2^, ^3^, ^4^ CCL is associated with serious complications, including acute obstructive suppurative cholangitis and pancreatitis.^5^, ^6^, ^7^ Hence, CCL management guidelines recommend lithotripsy for choledocholithiasis and cholecystectomy.^5^ In Japan, endoscopic retrograde cholangiopancreatography (ERCP) with laparoscopic cholecystectomy (LC) is the standard CCL treatment.^7^ Endoscopic biliary sphincterotomy and stone removal is the standard care for choledocholithiasis, and over 90% of all CBDS can be successfully treated via endoscopic sphincterotomy (EST) and stone extraction using baskets or balloon catheters.^8^ However, choledocholithiasis recurrence owing to remigrated gallstones is a concern during the waiting time for elective cholecystectomy. Using plastic stent placement as a bridge to surgery for the first therapy of CBDS is considered an alternative to initial stone removal treatment. Nevertheless, this strategy carries cholangitis risk owing to stent obstruction or migration while waiting for elective cholecystectomy. Regardless of which endoscopic treatment is performed, ERCP with LC is associated with the risk of acute cholecystitis and recurrent cholangitis while awaiting surgery. However, no previous prospective studies have reported the occurrence rate of acute cholecystitis or cholangitis, focusing on the waiting time for cholecystectomy, and the risk factors for these events remain unknown. Therefore, this retrospective study evaluated the incidence rate and risk factors for acute cholecystitis and cholangitis during elective cholecystectomy waiting time.

## MATERIALS AND METHODS

### Study design

We retrospectively analyzed consecutive patients who underwent ERCP and cholecystectomy for CCL between January 2019 and October 2021 at two tertiary care centers (Shizuoka General Hospital and Iwata City Hospital). We included patients who underwent cholecystectomy: (1) within 90 days after the first ERCP and (2) after 90 days but experienced biliary tract events within 90 days. Patients with acute cholecystitis during the first ERCP and plastic stent placement after lithotripsy were excluded.

Biliary tract events included acute cholecystitis, acute cholangitis, biloma, hepatic abscess, and stent trouble requiring re‐ERCP. The relevant institutional review board approved this retrospective study (approval number: SGHIRB#2022041), and the study adhered to the principles of the Declaration of Helsinki. Given the study's retrospective nature, the need for informed consent was waived.

### ERCP for choledocholithiasis

ERCP was performed using conventional side‐viewing scopes (JF‐260V, TJF‐260, TJF‐290V; Olympus Medical Systems). Endoscopic lithotripsy or biliary stenting was performed in asymptomatic patients with choledocholithiasis or mild cholangitis. Selective EST, endoscopic papillary balloon dilation , endoscopic papillary large balloon dilation, and treatment selection (stone removal before cholecystectomy or biliary stenting as the bridge to surgery) were performed at the discretion of the endoscopist. In principle, for asymptomatic CBDS or mild cholangitis patients, we prefer to choose to place a plastic stent alone to promote natural stone passage.^9^ However, the insertion of a plastic stent without EST increases the risk of PEP,^10^ and the final decision on whether to place a stent only, perform EST, or proceed to stone removal after EST is made by the attending physician, considering various factors such as the difficulty of the ERCP, the skill level of the endoscopist, whether the ERCP is elective or emergency, and patient factors. Regarding the selection of biliary stents, we used a diameter of 7 Fr. We utilized both pigtail and straight types of plastic stents, but there were no clear criteria for the selection of stent type. Initial biliary drainage was performed using nasobiliary drainage or plastic stent placement for patients with moderate or severe cholangitis. Stone removal was conducted after the patient's general condition improved, particularly if they required choledocholithotomy before cholecystectomy.

### Study outcomes and definitions

Patient information was obtained from each hospital's medical records and endoscopic databases. The CBD diameter was evaluated using the cholangiography of the first ERCP. The size and number of choledocholithiasis lesions were determined using endoscopic ultrasonography, magnetic resonance imaging, or computed tomography scans before the first ERCP. Furthermore, the size and number of gallstones were evaluated using abdominal ultrasonography or magnetic resonance imaging before the first ERCP. Acute cholecystitis and cholangitis were evaluated following the Tokyo Guidelines 2018,^7^ while other endoscopic adverse events (AEs) were evaluated based on the lexicon criteria.^11^ We evaluated post‐surgical AEs according to the International Study Group for Liver Surgery and the Clavien‐Dindo classification criteria.^12^, ^13^ Grade B and grade C bile leakage and grade IIIa or higher were defined as post‐surgical AEs. In this study, biliary tract events during the waiting period for elective cholecystectomy were defined as acute cholecystitis, acute cholangitis, or any unplanned ERCP due to choledocholithiasis or biliary stenting.

### Statistical analyses

Continuous variables were expressed as medians and interquartile ranges and analyzed using the Mann–Whitney U test. Categorical variables were presented as proportions and analyzed using Fisher's exact test. Univariate and multivariate analyses were performed to examine the risk factors for biliary tract events during the waiting period for elective cholecystectomy in all patients. The candidate factors included age, sex, endoscopic therapy for CBDS before cholecystectomy (stone removal before cholecystectomy or biliary stenting as a bridge to surgery), CBD diameter, number of CBDS, CBDS size, gallstone size, and EST. Factors with *p* < 0.20 in the univariate analysis were further assessed using a multivariate logistic regression analysis. All statistical tests were two‐tailed and assessed at a 0.05 probability level. All analyses were performed using the R version 3.4.1 (The R Foundation for Statistical Computing).

## RESULTS

During the study, 173 patients underwent cholecystectomy after initial choledocholithiasis therapy using ERCP (163 patients underwent cholecystectomy within 90 days after the first ERCP and 10 patients underwent cholecystectomy after 90 days but experienced biliary tract events within 90 days). Twelve patients were excluded from the analysis owing to simultaneous acute cholecystitis during the first ERCP or prophylactic bile duct stenting after lithotripsy. The remaining 151 patients were included in this study. Table 1 presents patient characteristics. The patients were categorized into biliary tract event and non‐biliary tract event groups (Table 1). No significant differences existed between both groups, except for CBDS therapy administered before cholecystectomy. Biliary tract events during the waiting period for cholecystectomy occurred more frequently in patients who underwent biliary stenting than in those who did not (*p* = 0.004).

**TABLE 1:** Baseline patient characteristics.

	All, *n* = 151	Biliary tract event group *n* = 43	Non‐biliary tract event group, *n* = 108	*p*‐value^†^
Age, median, (IQR)	69 (58.5–76)	67 (60–76)	70 (57.75–76)	0.58
Sex, male, *n* (%)	75 (50)	23 (53)	52 (48)	0.592
PS, 0/1/2/3/4, *n*	122/21/5/2/1	33/6/3/0/1	89/15/2/2/0	0.231
ASA‐PS, 1/2/3/4, *n*	47/80/23/1	13/20/10/0	34/60/13/1	0.337
Antithrombotic therapy agent, yes, *n* (%)	31 (21)	13 (30)	18 (17)	0.075
Maximum diameter of gallstones, *n*, median (IQR)	7 (5–10)	8 (5.5–11)	6 (4.75–10)	0.072
Number of CBDS, 1/2–4/≥5	95 (63)/47 (31)/9 (6)	31 (72)/10 (23)/2 (4.7)	64 (59)/37(34)/7(6.4)	0.32
Maximum diameter of CBDS, mm, median (IQR)	5 (4–7)	5 (4–8)	5 (4–7)	0.587
Maximum diameter of CBD, mm, median (IQR)	10 (8–12)	10 (9–12)	10 (8–12)	0.317
Preprocedural cholangitis, *n* (%)	78 (52)	24 (56)	54 (50)	0.59
Mild/moderate/severe	61/14/3	19/5/0	42/9/3	‐
Therapy for CBDS before cholecystectomy				
Endoscopic lithotripsy/biliary stenting^‡^, *n* (%)	51 (34)/100 (66)	7 (16)/36 (84)	44 (41)/64 (59)	0.004
The type of biliary stent, pigtail/ straight	69/ 31	27/ 9	42/ 22	0.375
Procedure for Varter's papilla	85 (56)	21 (49)	64 (59)	0.278
EST/EPBD/EPLBD	81/1/3	21/0/0	60/1/3	‐

^†^ Compared to the biliary event group and non‐biliary event group

^‡^ Endoscopic lithotripsy was planned after the cholecystectomy during the first ERCP.

Abbreviations: ASA‐PS, American Society of America Anesthesiologists physical status; CBD, common bile duct; CBDS, common bile duct stone; EPBD, endoscopic papillary balloon dilation; EPLBD, endoscopic papillary large balloon dilation; EST, endoscopic sphincterotomy; IQR, interquartile range; PS, performance status.

Table 2 displays biliary tract events during the waiting time for cholecystectomy. The median waiting time was 47 days. Among the patients, 30 experienced acute cholecystitis, 15 had cholangitis, and 14 required unplanned ERCP. Operation time was longer in the biliary tract events group than in the non‐biliary tract events group. Post‐surgical AEs were similar between the two groups.

**TABLE 2:** Biliary tract events during the cholecystectomy waiting time.

	*n* = 151	Biliary tract event group, *n* = 43	Non‐biliary tract event group, *n* = 108	*p*‐value
Waiting time before surgery, days, median (IQR)	47 (28–64.5)	47 (21.5–79.5)	46.5 (30.75–64)	0.729
Biliary tract events during the waiting time before surgery, *n* (%)	43 (28)	43 (28)	‐	‐
Acute cholecystitis, *n* (%)	30 (20)	30 (20)	‐	‐
Acute cholangitis, *n* (%)	15 (11)	15 (11)	‐	‐
Due to the recurrence of CBDS, *n*	2	2	‐	‐
Due to stent obstruction/migration, *n*	13	13	‐	‐
Pain due to biliary stent placement, *n* (%)	1 (0.7)	1 (0.7)	‐	‐
Unplanned ERCP during the waiting time before surgery, *n* (%)	14 (9.2)	14 (9.2)	‐	‐
Cholecystectomy				
Laparoscopic/open^†^, *n* (%)	131 (87)/20 (13)	36 (84)/7 (16)	95 (88)/13 (12)	0.595
Surgery time, minute, mean (IQR)	119 (94.5‐152.5)	139 (104‐164)	113 (93‐143)	0.041
Post‐surgical adverse events, *n* (%)	12 (7.9)	2 (4.7)	10 (9.3)	0.51
SSI, *n*	10	1	9	‐
Bleeding, *n*	1	0	1	‐
Perforation of the duodenum, *n*	1	1	0	‐

^†^ Including transition from laparoscopic surgery to open surgery.

Abbreviations: CBDS, common bile duct stone; ERCP, endoscopic retrograde cholangiopancreatography; IQR, interquartile range; SSI, surgical site infection.

### Risk factors for biliary tract events during the waiting time for cholecystectomy

Table 3 presents the results of univariate and multivariate analyses of the risk factors for Recurrent Biliary Obstruction. Based on the multivariate analysis, plastic stent placement as a bridge to surgery for the first CBDS therapy was an independent risk factor for biliary tract events during the waiting time for surgery (odds ratio [OR]: 4.74, p = 0.002).

**TABLE 3:** Risk factors for biliary tract events during the preoperative waiting time.

		Univariate			Multivariate		
Factors		**OR**	**95% CI**	***p*‐value**	**OR**	**95% CI**	***p*‐value**
Therapy for CBDS before	PS placement	4.25	1.72–10.5	0.002	3.59	1.45–8.89	0.006
cholecystectomy	Lithotripsy	1			1		
Age	> 60	0.97	0.45–2.10	0.942			
	≦ 60	1					
Sex	Male	1.17	0.57–2.42	0.548			
	Female	1					
Antithrombotic agent	Yes	2.17	0.95–4.94	0.066	2.23	0.94–5.24	0.07
	No	1					
Common bile duct diameter	≦ 10 mm	1.11	0.53–2.32	0.858			
	> 10 mm	1					
Number of CBDS	≥ 2	0.56	025–1.21	0.725			
	1	1					
Maximum diameter of CBDS	> 5 mm	1.50	0.74–3.06	0.264			
	≦ 5 mm	1					
Maximum diameter of gallstones	≤ 5 mm	0.82	0.38–1.79	0.268			
	> 5 mm	1					
EST/EPBD/EPLBD	Yes	0.53	0.26–1.10	0.318			
	No	1					

Abbreviations: CBDS, common bile duct stone; CI, confidence interval; EPBD, endoscopic papillary balloon dilation; EPLBD, endoscopic papillary large balloon dilation; EST, endoscopic sphincterotomy; OR, odds ratio.

### Comparison of endoscopic lithotripsy and plastic stent placement before cholecystectomy

Table 4 compares patient characteristics between both groups (endoscopic lithotripsy before cholecystectomy and plastic stent placement as a bridge to surgery). Except for CBDS size and procedure for Vater papilla (including EST, endoscopic papillary balloon dilation, and endoscopic papillary large balloon dilation), baseline patient characteristics were similar between the two groups. The endoscopic lithotripsy group included patients with large CBDS, and EST was more frequently performed in this group. The waiting time for cholecystectomy was similar in both groups (Table 5). Table 5 also summarizes the biliary tract events. The rate of biliary tract events before cholecystectomy was significantly higher in the plastic stent placement group than in the endoscopic lithotripsy group (*p* = 0.004).

**TABLE 4:** Comparison of baseline patient characteristics between the endoscopic lithotripsy and stent placement groups.

	Endoscopic lithotripsy before cholecystectomy *n* = 51	Plastic stent placement as a bridge to surgery *n* = 100	*p*‐value
Age, median, (IQR)	71 (63.5–77)	66 (55–74.25)	0.061
Sex, male, *n* (%)	27 (53)	48 (48)	0.608
PS, 0/1/2/3/4, *n*	37/11/2/0/1	85/10/3/2/0	0.112
ASA‐PS, 1/2/3/4, *n*	13/31/6/1	34/49/17/0	0.224
Antithrombotic therapy agent, yes, *n* (%)	10 (20)	21 (21)	1
Maximum diameter of gallstones, mm, median (IQR)	8 (4–15)	6 (5–10)	0.347
Number of CBDS, 1/2–4/≥5	33 (65)/15 (29)/3 (6)	62 (62)/32 (32)/6 (6)	0.958
Maximum diameter of CBDS, mm, median (IQR)	6 (4.5–8)	5 (4–6)	0.011
Maximum diameter of CBD, mm, median (IQR)	11 (8.5–12)	10 (8–12)	0.119
Preprocedural cholangitis, *n* (%)	25 (49)	53 (53)	0.731
Mild/moderate/severe	47/3/1	87/11/2	‐
Procedure for Vater's papilla, yes, *n* (%)	51 (100)	35 (35)	< 0.001
EST/EPBD/EPLBD	47/1/3	35/0/0	‐

Abbreviations: ASA‐PS, American Society of America Anesthesiologists physical status; CBDS, common bile duct stone; EPBD, endoscopic papillary balloon dilation; EPLBD, endoscopic papillary large balloon dilation; EST, endoscopic sphincterotomy; PS, performance status.

**TABLE 5:** Comparison of biliary tract events during the waiting time of cholecystectomy between endoscopic lithotripsy and stent placement.

	Endoscopic lithotripsy before cholecystectomy, *n* = 51	Plastic stent placement as a bridge to surgery, *n* = 100	*p*‐value
Waiting time before surgery, days, median (IQR)	44 (30.5–64.5)	47.5 (27–65)	0.875
Biliary tract events during the waiting time before surgery, *n* (%)	7 (14)	36 (36)	0.004
Acute cholecystitis, *n* (%)	6 (12)	24 (24)	0.087
Acute cholangitis, *n* (%)	2 (3.9)	13 (13)	0.091
Owing to the recurrence of CBDS, *n*	2	‐	‐
Owing to stent obstruction/migration, *n*	‐	10/3	‐
Pain due to biliary stent placement, *n* (%)	‐	1 (1)	‐
Time to biliary tract events from ERCP, days, median (IQR)	26 (21.5–29.5)	15 (6.75–38.5)	‐
Unplanned ERCP during the waiting time before surgery, *n* (%)	2 (3.9)	12 (12)	0.141

Abbreviations: CBDS, common bile duct stone; ERCP, endoscopic retrograde cholangiopancreatography; IQR, interquartile range.

### Risk factors for acute cholecystitis and cholangitis in patients who underwent plastic stent placement during the waiting time for cholecystectomy

A subgroup analysis of patients who underwent plastic stent placement uncovered a CBD diameter of ≤ 10 mm as an independent risk factor for acute cholecystitis (Table 6, OR: 5.37, p = 0.015). Conversely, a CBD diameter of c 11 mm was an independent risk factor for acute cholangitis (Table 7, OR: 4.23, p = 0.039) in the univariate and multivariate analysis.

**TABLE 6:** Risk factors for acute cholecystitis during the waiting period for surgery in patients with stent placement.

		Univariate			Multivariate		
Factors		**OR**	**95% CI**	***p*‐value**	**OR**	**95% CI**	***p*‐value**
PS type	Pigtail	0.87	0.33–2.31	0.777			
	Straight	1					
Age	> 60	0.69	0.27–1.76	0.433			
	≦ 60	1					
Sex	Male	1.38	0.55–3.48	0.489			
	Female	1					
Antithrombotic agent	Yes	2.42	0.858–6.84	0.094	3.26	1.05–10.1	0.041
	No	1					
Common bile duct	≤10 mm	4.32	1.18–15.8	0.027	5.37	1.39–20.8	0.015
diameter	>10 mm	1			1		
Number of CBDS	≥2	0.97	0.38–2.51	0.954			
	1	1					
Maximum diameter of	>5 mm	1.55	0.60–3.98	0.365			
CBDS	≤5 mm	1					
Maximum diameter of	≤5 mm	0.55	0.19–1.57	0.264			
gallstones	>5 mm	1					
EST/EPBD/EPLBD	Yes	0.91	0.34–2.39	0.844			
	No	1					

Abbreviations: CBDS, common bile duct stone; CI, confidence interval; EPBD, endoscopic papillary balloon dilation; EPLBD, endoscopic papillary large balloon dilation; EST, endoscopic sphincterotomy; OR, odds ratio; PS, plastic stent.

**TABLE 7:** Risk factors for acute cholangitis during the waiting period for surgery in patients with stent placement.

Factors		Univariate			Multivariate		
		**OR**	**95% CI**	***p*‐value**	**OR**	**95% CI**	***p*‐value**
PS type	Pigtail	6.32	0.78–50.9	0.084	5.54	0.62–49.8	0.126
	straight	1			1		
Age	>60	1.25	0.35–4.38	0.732			
	≤60	1					
Sex	Male	1.88	0.57–6.21	0.300			
	Female	1					
Antithrombotic agent	Yes	4.11	1.21–14	0.024	2.92	0.74–11.5	0.125
	No	1					
Common bile duct	10 mm	6.26	1.76–22.3	0.005	4.23	1.08–16.7	0.039
diameter	≤10 mm	1			1		
Number of CBDs	≥2	0.45	0.11–1.74	0.244			
	1	1					
Maximum diameter of	>5 mm	5.58	1.57–19.8	0.008	3.55	0.89–14.1	0.072
CBDS	≤5 mm	1					
Maximum diameter of	≤5 mm	1.54	0.31–7.54	0.596			
gallstones	>5 mm	1					
EST/EPBD/EPLBD	Yes	1.19	0.36–3.95	0.779			
	No	1					

Abbreviations: CBDS, common bile duct stone; CI, confidence interval; EPBD, endoscopic papillary balloon dilation; EPLBD, endoscopic papillary large balloon dilation; EST, selective endoscopic sphincterotomy; OR, odds ratio; PS, plastic stent.

## DISCUSSION

Cholecystectomy after ERCP is a widely accepted therapy for CCL; however, investigations on the incidence of biliary tract events and their associated risk factors are limited. This retrospective study evaluated the risk factors of acute cholecystitis and acute cholangitis during the waiting time for elective cholecystectomy after the first ERCP in patients with CCL. Our findings revealed that biliary plastic stent placement was a risk factor for biliary tract events during the preoperative waiting period. Furthermore, a large and narrow CBD was a risk factor for acute cholangitis and cholecystitis, respectively, in patients who underwent plastic placement as a bridge to surgery.

No previous prospective studies have reported the occurrence rate of acute cholecystitis or cholangitis, focusing on the waiting time for cholecystectomy. The guidelines from the European Society of Gastrointestinal Endoscopy recommend performing LC within 2 weeks following ERCP for choledocholithiasis to reduce the risk for future biliary‐related disease.^14^, ^15^ However, in clinical practice, performing cholecystectomy within 2 weeks is often difficult due to several social factors or patient preferences. LC and laparoscopic CBD exploration (LCBDE) are treatment options for CCL. The safety and efficacy of LC+LCBDE have been reported.^16^ However, LC+LCBDE also has disadvantages, such as a higher rate of bile leakage, electrolyte disturbance, and reduced quality of life due to T‐tube retention.^16^ The latest consensus guidelines, including those established by the European Society of Gastrointestinal Endoscopy and the American Society for Gastrointestinal Endoscopy,^14^, ^15^, ^17^ indicate insufficient evidence to establish the best approach for CCL. Endoscopic lithotripsy and LC are often performed for CCL. Therefore, knowing the risk factors for biliary‐related diseases during the waiting time for cholecystectomy is essential, leading to the early treatment of affected individuals.

Based on the findings of this retrospective study, the incidence of biliary‐related disease during the waiting time for cholecystectomy was 28% (43 cases), with incidence rates of 20% and 11% for acute cholecystitis and cholecystitis, respectively. Furthermore, plastic stent placement as a bridge to surgery for the first CBDS was an independent risk factor for biliary tract events during the preoperative waiting time. Not only the waiting time for cholecystectomy, retrospective and prospective series have reported biliary complications in 4%–24% of patients after varying periods of follow‐up, and the subsequent cholecystectomy rate is 5.8%–18%.^18^, ^19^, ^20^, ^21^, ^22^, ^23^, ^24^ The inclusion criterion of our study could potentially inflate the apparent rates of AEs. Specifically, patients who initially did not want to undergo cholecystectomy but decided to do so because an event occurred may be overrepresented in the event group. Furthermore, this study included only patients with CCL; thus, the rate of biliary tract events may be higher than in past reports.

Endoscopic removal of CBDS carries a risk of choledocholithiasis recurrence due to gallstone removal. However, the rate of acute cholangitis due to choledocholithiasis recurrence was low (3.9%) during the waiting time for cholecystectomy. Nonetheless, plastic stent placement as a bridge to surgery carries the risk of acute cholangitis due to stent‐related complications (stent obstruction or migration), with a cholangitis rate of 13%. Giorgio et al. reported that cholangitis developed in 7.8% of patients during the 3 months after plastic stent placement, and the risk of cholangitis increased with the length of stent placement time.^25^ Early cholecystectomy and biliary stent removal are recommended in cases involving bile duct stenting because of the increased incidence of cholangitis.

While the risk of biliary‐related events was higher in patients undergoing biliary stent placement, biliary stenting has several benefits as a bridge to surgery. Recent studies have revealed that most CBDS decrease in size following stenting, with the possibility of complete stone elimination.^9^, ^26^, ^27^ This suggests that CBDS can be treated using biliary stent placement while preserving the function of the Vater papilla. This approach is particularly critical in young patients. Biliary stenting treatment must also be chosen when immediate decisions about withdrawing anticoagulants or antiplatelet agents are challenging. Therefore, understanding the risk factors for stent‐related events in patients undergoing biliary stent placement is crucial. Based on our data, narrowing the CBD is an independent risk factor for acute cholecystitis, and a large CBD is an independent risk factor for acute cholangitis. It is well‐known that MS, particularly covered metal stents, are associated with a higher risk of cholecystitis ^28^ and Noguchi et al. identified a thin bile duct diameter as a significant risk factor for acute cholecystitis in patients with covered self‐expandable metal stent placement.^29^ On the other hand, Ting et al. and Kim et al. reported that the risk of cholecystitis post‐endoscopic biliary drainage (EBD) was not influenced by the type of EBD used,^30^, ^31^ suggesting that biliary plastic stents themselves might carry an inherent risk of cholecystitis. As the mechanism of cholecystitis onset, factors such as the impact on the orifice of the cystic duct, secondary bile flow obstruction in the gallbladder due to stent occlusion, infection triggered by retrograde intestinal bacteria, and cystic duct contrast can be considered.^28^, ^30^, ^31^, ^32^ In narrower bile ducts, these effects may be more pronounced, thus it is necessary to be vigilant for cholecystitis after bile duct stent placement without CBDS removal.

Additionally, a large CBD was identified as an independent risk factor for acute cholangitis. Sugiyama et al. and Sujuan et al. reported that a large CBD diameter is an independent risk factor for choledochal complications.^33^, ^34^ Regarding the risk of recurrent cholangitis after the treatment of choledocholithiasis, a large CBD is known to be a risk factor.^35^ These are suggested to be due to the association between bile stasis and a large CBD.^35^ In patients with a large CBD, bile tends to stagnate, which may facilitate the formation of biliary sludge. This sludge formation can increase the likelihood of early obstruction of biliary stents, thereby leading to cholangitis. Thus, Patients with a large CBD need to be aware of cholangitis during the cholecystectomy waiting time.

The limitations of our study must be acknowledged when interpreting the results. First, our study had a retrospective, two‐center design. Therefore, the optimal indications for cholecystectomy remain unclear. Not all patients with CCL undergo cholecystectomy. However, the strength of our study is that it included only patients who underwent cholecystectomy, which is suitable for assessing the risk factors during cholecystectomy. Second, although the indications for the treatment strategy of CBDS have been described, this is a retrospective study, and the optimal indications for choosing between bile duct stent placement and stone removal also remain unclear. Our results showed no significant differences without the Maximum diameter of CBDS in patient backgrounds and we analyzed the risk factors for biliary events using univariate and multivariate analyses. However, prospective observational studies are considered necessary. Thirdly, there are no clear indications for the selection criteria of the type of plastic stent. Although no differences in outcomes were observed based on the type of stent in this study, the possibility that the type of stent could influence the outcomes cannot be ruled out, suggesting that studies using the same specifications may also be necessary.

In conclusion, plastic stent placement for CBDS before elective cholecystectomy is associated with a risk of acute cholecystitis or acute cholangitis during the waiting time for elective cholecystectomy. It is advisable to remove CBDS during the initial ERCP to reduce the risk of biliary complications while awaiting surgery.

## CONFLICT OF INTEREST STATEMENT

None.

## ETHICS STATEMENT

Approval of the research protocol by an Institutional Reviewer Board: The relevant institutional review board approved this retrospective study (approval number: SGHIRB#2022041).

## PATIENT INFORMED CONSENT

N/A

## Data Availability

The data presented in this study are available on request from the corresponding author. The data are not publicly available due to privacy concerns.
